# 53BP1 loss rescues embryonic lethality but not genomic instability of BRCA1 total knockout mice

**DOI:** 10.1038/s41418-020-0521-4

**Published:** 2020-03-05

**Authors:** Jiyuan Chen, Peng Li, Licun Song, Long Bai, Michael S. Y. Huen, Yidan Liu, Lin-Yu Lu

**Affiliations:** 1 Key Laboratory of Reproductive Genetics (Ministry of Education) and Women’s Reproductive Health Laboratory of Zhejiang Province, Women’s Hospital, Zhejiang University School of Medicine, Hangzhou, China; 2 Institute of Translational Medicine, Zhejiang University School of Medicine, Hangzhou, China; 3 School of Biomedical Sciences, LKS Faculty of Medicine, The University of Hong Kong, Hong Kong SAR, China

**Keywords:** Cell biology, Cell biology, Cancer genetics, Cancer genetics

## Abstract

BRCA1 is critical for DNA double-strand break (DSB) repair by homologous recombination (HR). BRCA1 deficient mice are embryonic lethal. Previous studies have shown that 53BP1 knockout (KO) rescues embryonic lethality of BRCA1 hypomorphic mutant mice by restoring HR. Here, we show that 53BP1 KO can partially rescue embryonic lethality of BRCA1 total KO mice, but HR is not restored in BRCA1-53BP1 double knockout (DKO) mice. As a result, BRCA1-53BP1 DKO cells are extremely sensitive to PARP inhibitors (PARPi). In addition to HR deficiency, BRCA1-53BP1 DKO cells have elevated microhomology-mediated end joining (MMEJ) activity and G2/M cell cycle checkpoint defects, causing severe genomic instability in these cells. Interestingly, BRCA1-53BP1 DKO mice rapidly develop thymic lymphoma that is 100% penetrant, which is not observed in any BRCA1 mutant mice rescued by 53BP1 KO. Taken together, our study reveals that 53BP1 KO can partially rescue embryonic lethality caused by complete BRCA1 loss without rescuing HR-related defects. This finding suggests that loss of 53BP1 can support the development of cancers with silenced BRCA1 expression without causing PARPi resistance.

## Introduction

Cells in our body constantly encounter all kinds of DNA damage that are repaired by different DNA damage repair pathways. DNA double-strand break (DSB) is one of the most deleterious forms of DNA damage. Homologous recombination (HR) is an important pathway for DSB repair that requires loading of key enzyme RAD51 to DSBs for repair using sister chromatids as templates [1]. HR deficiency impairs the maintenance of genomic stability and often leads to tumorigenesis [2].

BRCA1 is a key protein for HR whose loss or mutation leads to HR deficiency and genomic instability [3]. BRCA1 mutations are frequently identified in familial breast and ovarian cancers [4]. BRCA1 loss or mutation leads to PARP inhibitor (PARPi) sensitivity, which has been exploited to treat BRCA1 mutant cancers [5]. Besides tightly linked to tumorigenesis, HR is also important for embryonic development. BRCA1 deficient mice are embryonic lethal [6], but the exact role of BRCA1 in embryonic development is not clear.

In the past decades, studies have provided mechanistic insights into how BRCA1 promotes HR. BRCA1 interacts with and recruits PALB2 to DSBs, which is essential for BRCA2/RAD51 loading [7–9]. Besides HR, DSB can be repaired by nonhomologous end joining (NHEJ). BRCA1 also functions in DSB repair pathway choices between HR and NHEJ [10, 11]. BRCA1 removes 53BP1 from DSB ends and promotes DNA end resection, a prerequisite for HR. It is believed that 53BP1 KO lifts the barrier on DNA end resection and restores HR in the absence of intact BRCA1 [12–15].

**Fig. 1:**
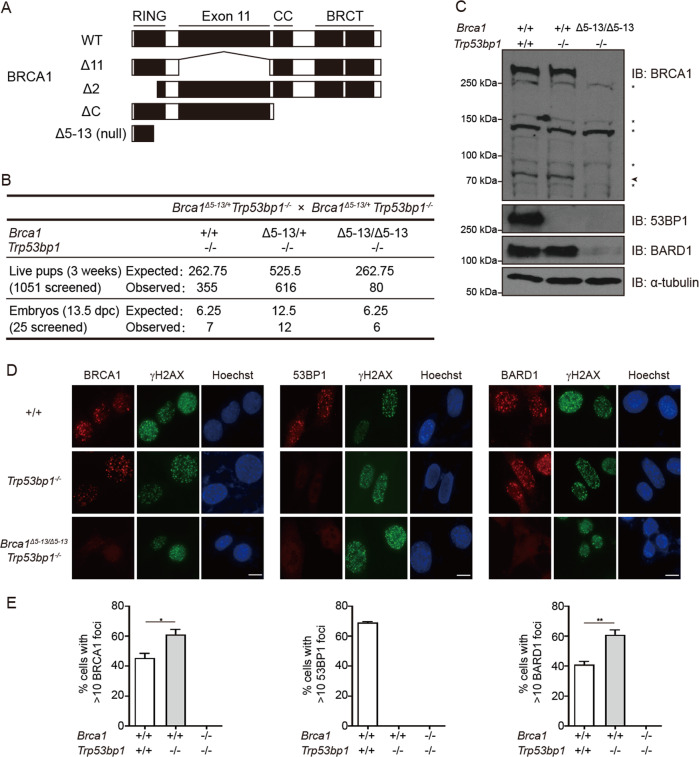
53BP1 KO rescues embryonic lethality of BRCA1 KO mice. **a** Domain structures of BRCA1 WT, Δ11, Δ2, ΔC, and Δ5–13 proteins. CC coiled coil. **b** Summary of mice at 3 weeks or embryos at 13.5 dpc of indicated genotypes. **c** Western blotting analyses of BRCA1, 53BP1, and BARD1 protein in MEFs with indicated genotypes. α-tubulin was used as loading control. Asterisk, nonspecific band. Arrowhead, BRCA1 isoforms or degraded BRCA1 proteins. **d** Immunofluorescence staining analyses of BRCA1, 53BP1, and BARD1 foci in MEFs with indicated genotypes 6 h after 10 Gy IR exposure. γH2AX marks sites of DNA damage. Hoechst 33342 marks nucleus. Scale bars, 10 μm. **e** Quantification of BRCA1, 53BP1, and BARD1 foci. Error bars represent SEM from three independent experiments. *p < 0.05; **p < 0.01.

The identification of BRCA1’s function in DSB repair pathway choice originates from the finding that 53BP1 KO can fully rescue the embryonic lethality of *Brca1*^*Δ11/Δ11*^ and *Brca1*^*Δ2/Δ2*^ mice [12–15]. The HR deficiency and genomic instability are also fully rescued so that both *Brca1*^*Δ11/Δ11*^*;Trp53bp1*^*−/−*^ mice and *Brca1*^*Δ2/Δ2*^*;Trp53bp1*^*−/−*^ mice have normal life span and no elevated tumor incidences than wild-type (WT) mice [12–15]. However, both *Brca1*^*Δ11/Δ11*^ and *Brca1*^*Δ2/Δ2*^ mice are *Brca1* hypomorphic mutant but not total KO mice. In these mice, truncated BRCA1Δ11 and BRCA1Δ2 proteins are produced, both of which retain the BRCT domain that localizes the protein to DSBs and the coiled coil domain that interacts with PALB2 (Fig. 1a). A recent study has shown that 53BP1 KO can also fully rescue embryonic lethality of *Brca1*^*ΔC/ΔC*^ mice [16]. Interestingly, HR deficiency and genomic instability are only mildly rescued in *Brca1*^*ΔC/ΔC*^*;Trp53bp1*^*−/−*^ mice and these mice have shorter life span [16]. Theoretically, a truncated BRCA1ΔC protein that deletes both the BRCT and the coiled coil domain is produced in *Brca1*^*ΔC/ΔC*^ mice, but no truncated protein could be detected, suggesting that *Brca1*^*ΔC/ΔC*^ mice are close to BRCA1 total KO mice. However, it is still possible that BRCA1 is not completely absent in *Brca1*^*ΔC/ΔC*^ mice, in which the truncated BRCA1ΔC proteins might express at low levels and contribute to the viability of *Brca1*^*ΔC/ΔC*^*;Trp53bp1*^*−/−*^ mice. Therefore, it remains to be studied if 53BP1 KO can rescue the lethality of *bona fide* BRCA1 total KO mice.

In this study, we report that 53BP1 KO can partially rescue embryonic lethality of *bona fide* BRCA1 total KO mice, but HR deficiency is not restored. BRCA1-53BP1 double knockout (DKO) mice have severe genomic instability and G2/M cell cycle checkpoint defects and develop a unique type of thymic lymphoma that is 100% penetrant. This study reveals that HR deficiency is compatible with embryonic development, but HR is important for preventing tumorigenesis in adults.

## Results

### 53BP1 KO partially rescues embryonic lethality of BRCA1 total KO mice

To investigate if 53BP1 KO can rescue embryonic lethality of BRCA1 total KO mice, we utilized mice homozygous for a *Brca1* null allele that deletes exons 5–13 [17]. Unlike BRCA1Δ11, BRCA1Δ2, or BRCA1ΔC proteins, no intact domains are left for BRCA1Δ5*–*13 protein (Fig. 1a). We generated mice heterozygous for *Brca1* null allele in *Trp53bp1*^*−/−*^ background (*Brca1*^*Δ5–13/+*^*;Trp53bp1*^*−/−*^) and intercrossed them. Genotyping of new born mice at 3 weeks revealed some viable *Brca1*^*Δ5–13/Δ5–13*^*;Trp53bp1*^*−/−*^ mice, but they were obtained at a lower Mendelian ratio (Fig. 1b). *Brca1*^*Δ5–13/Δ5–13*^*;Trp53bp1*^*−/−*^ embryos were obtained with normal Mendelian ratio at 13.5 days post coitum (dpc) (Fig. 1b), suggesting that some embryos die afterwards. Examination of *Brca1*^*Δ5–13/Δ5–13*^*;Trp53bp1*^*−/−*^ mouse embryonic fibroblasts (MEFs) confirmed the absence of BRCA1 and 53BP1 proteins (Fig. 1c). Consistent with previous observations that BRCA1 forms a heterodimer with BARD1 and is required for its stability [18, 19], a dramatic reduction of BARD1 protein level was observed in *Brca1*^*Δ5–13/Δ5–13*^*;Trp53bp1*^*−/−*^ MEFs (Fig. 1c). Ionizing radiation-induced foci of BRCA1, 53BP1, or BARD1 could not be observed in *Brca1*^*Δ5–13/Δ5–13*^*;Trp53bp1*^*−/−*^ MEFs either (Fig. 1d, e). Therefore, *Brca1*^*Δ5–13*^ allele is a *bona fide Brca1* null allele and will be referred to as *Brca1*^*−*^ allele hereinafter. These observations suggest that 53BP1 KO can partially rescue the embryonic lethality of BRCA1 total KO mice.

### HR efficiency is not restored in BRCA1-53BP1 DKO cells

**Fig. 2:**
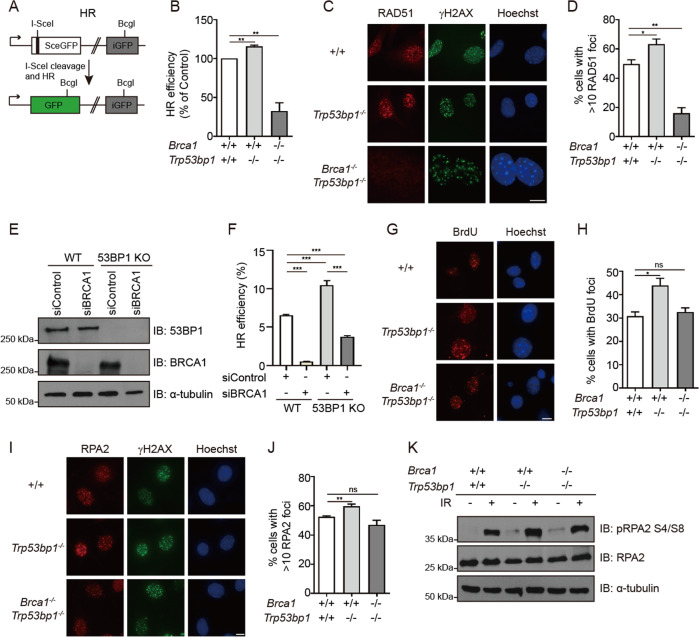
HR efficiency is not restored in BRCA1-53BP1 DKO cells. **a** Schematic representation of the DR-GFP reporter for analyzing efficiency of DSB repair by HR. **b** Summary of HR efficiency obtained using the DR-GFP reporter in MEFs with indicated genotypes. **c** Immunofluorescence staining analyses of RAD51 foci in MEFs with indicated genotypes 6 h after 10 Gy IR exposure. γH2AX marks sites of DNA damage. Hoechst 33342 marks nucleus. Scale bars, 10 μm. **d** Quantification of RAD51 foci. **e** Western blotting analyses of BRCA1 and 53BP1 protein in WT and 53BP1 KO U2OS DR-GFP cells after control or BRCA1 knockdown. α-tubulin was used as loading control. **f** Summary of HR efficiency in WT and 53BP1 KO U2OS DR-GFP cells after control or BRCA1 knockdown. **g** MEFs with indicated genotypes were labeled with 10 μM BrdU and 24 h later cells were treated with 10 Gy IR and recovered for 1 h. Representative BrdU foci were shown. Hoechst 33342 marks nucleus. Scale bars, 10 μm. **h** Quantification of BrdU foci. **i** Immunofluorescence staining analyses of RPA2 foci in MEFs with indicated genotypes 6 h after 10 Gy IR exposure. γH2AX marks site of DNA damage. Hoechst 33342 marks nucleus. Scale bars, 10 μm. **j** Quantification of RPA2 foci. **k** Western blotting analyses of RPA2 and RPA2 phosphorylation (S4/8) in MEFs with indicated genotypes 1 h after 10 Gy IR exposure. α-tubulin was used as loading control. Error bars represent SEM from three independent experiments. *p < 0.05; **p < 0.01; ***p < 0.001; ns not significant.

Previous studies have shown that 53BP1 KO fully rescues HR defects in *Brca1*^*Δ11/Δ11*^ and *Brca1*^*Δ2/Δ2*^ cells [12–15]. Interestingly, severe HR defects were still observed in *Brca1*^*−/−*^*;Trp53bp1*^*−/−*^ MEFs using a plasmid-based HR reporter (Fig. 2a, b). Consistent with HR defects, ionizing radiation-induced RAD51 foci was significantly reduced in *Brca1*^*−/−*^*;Trp53bp1*^*−/−*^ MEFs (Fig. 2c, d). To confirm this observation, BRCA1 was depleted by siRNA in U2OS cells stably integrated with an HR reporter to mimic BRCA1 status in *Brca1*^*−/−*^ cells (Fig. 2e, f). In this system, 53BP1 KO could only partially restore the HR defect caused by BRCA1 depletion (Fig. 2f). These experiments collectively suggest that, unlike *Brca1*^*Δ11/Δ11*^ or *Brca1*^*Δ2/Δ2*^ cells but similar to *Brca1*^*ΔC/ΔC*^ cells, 53BP1 KO cannot rescue the HR defects caused by complete BRCA1 loss.

In *Brca1*^*Δ11/Δ11*^, *Brca1*^*Δ2/Δ2*^, and *Brca1*^*ΔC/ΔC*^ cells, 53BP1 KO fully restores DNA end resection [13, 14, 16]. Similarly, no defect in DNA end resection was observed in *Brca1*^*−/−*^*;Trp53bp1*^*−/−*^ MEFs since ionizing radiation-induced BrdU foci, RPA2 foci and RPA2 phosphorylation were normal (Fig. 2g–k). It is likely that 53BP1 KO can fully rescue DNA end resection defects but not HR defects caused by complete BRCA1 loss.

### Genomic instability is not restored in BRCA1-53BP1 DKO cells

**Fig. 3:**
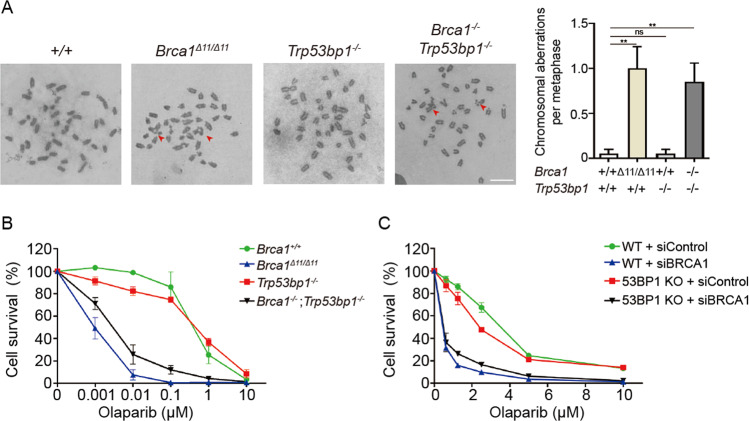
Genomic instability is not restored in BRCA1-53BP1 DKO cells. **a** Summary of chromosomal aberration numbers per metaphase in ES cells with indicated genotypes after overnight treatment of 1 μM PARPi olaparib. Arrows show examples of chromosome aberrations. Scale bars, 10 μm. **b** Cell viability analyses of ES cells with indicated genotypes after treatment with indicated doses of olaparib. **c** Cell viability analyses of WT and 53BP1 KO U2OS DR-GFP cells after control or BRCA1 knockdown and treatment with indicated doses of olaparib. Error bars represent SEM from three independent experiments.

Due to HR deficiency, *Brca1*^*Δ11/Δ11*^, *Brca1*^*Δ2/Δ2*^, and *Brca1*^*ΔC/ΔC*^ cells have severe genomic instability. They are hypersensitive to PARPi due to the presence of massive chromosomal aberrations after PARPi treatment. Since 53BP1 KO fully rescued PARPi sensitivity in *Brca1*^*Δ11/Δ11*^ and *Brca1*^*Δ2/Δ2*^ cells but not in *Brca1*^*ΔC/ΔC*^ cells [13–16], we examined if 53BP1 KO could rescue the PARPi sensitivity caused by complete BRCA1 loss. As *Brca1*^*−/−*^ MEFs could not be obtained for direct comparison, we performed PARPi sensitivity assay using *Brca1*^*−/−*^*;Trp53bp1*^*−/−*^ embryonic stem (ES) cells and used *Brca1*^*Δ11/Δ11*^ ES cells for comparison. After acute PARPi treatment, both *Brca1*^*−/−*^*;Trp53bp1*^*−/−*^ and *Brca1*^*Δ11/Δ11*^ ES cells had significant numbers of chromosome aberrations, which were barely present in WT or *Trp53bp1*^*−/−*^ ES cells (Fig. 3a). Consistently, *Brca1*^*−/−*^*;Trp53bp1*^*−/−*^ ES cells were only slightly less sensitive to PARPi than *Brca1*^*Δ11/Δ11*^ ES cells and were much more sensitive than WT or *Trp53bp1*^*−/−*^ ES cells (Fig. 3b). We also performed PARPi sensitivity assay in WT and 53BP1 KO U2OS cells after depletion of BRCA1 by siRNA. BRCA1 depletion in 53BP1 KO cells still lead to PARPi sensitivity (Fig. 3c). These experiments suggest that 53BP1 KO cannot rescue the genomic instability and PARPi sensitivity caused by complete BRCA1 loss.

### BRCA1-53BP1 DKO mice develop thymic lymphoma

**Fig. 4:**
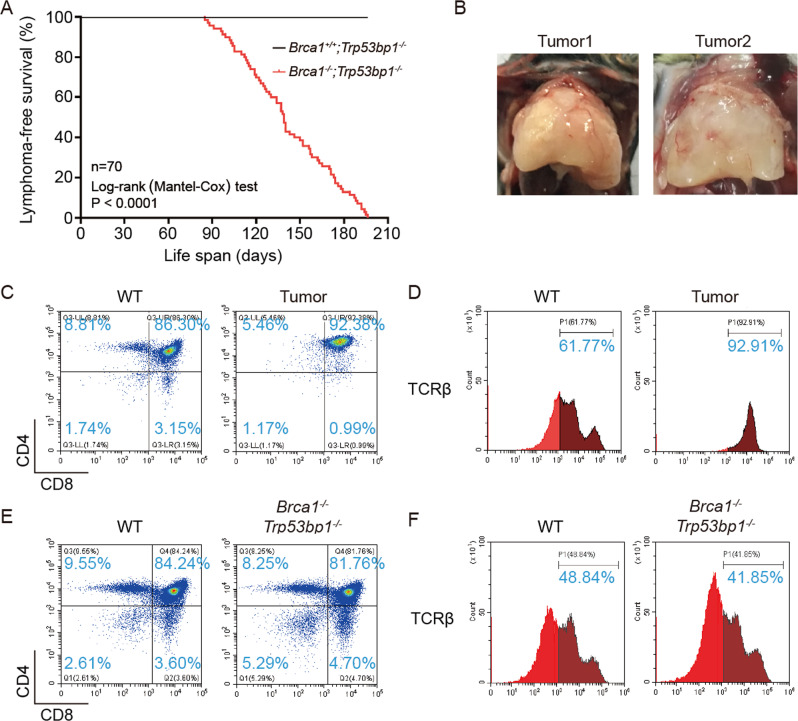
BRCA1-53BP1 DKO mice develop thymic lymphoma. **a** Summary of lymphoma-free survival of mice with indicated genotypes over 7 months using the Kaplan–Meier method. **b** Representative images of thymic lymphomas from BRCA1-53BP1 DKO mice. Representative flow cytometry analyses of CD4/CD8 (**c**) and TCRβ (**d**) surface expression in lymphoma cells from BRCA1-53BP1 DKO mice using thymocytes from 8-week-old WT mice as control. Representative flow cytometry analyses of CD4/CD8 (**e**) and TCRβ (**f**) surface expression in thymocytes from 1-month-old WT and BRCA1-53BP1 DKO mice.

Genomic instability is tightly linked to tumor formation. *Brca1*^*Δ11/Δ11*^*;Trp53bp1*^*−/−*^ and *Brca1*^*Δ2/Δ2*^*;Trp53bp1*^*−/−*^ mice are indistinguishable from WT mice in terms of life span or tumor incidence [12, 15]. Similar to *Brca1*^*ΔC/ΔC*^*;Trp53bp1*^*−/−*^ mice, *Brca1*^*−/−*^*;Trp53bp1*^*−/−*^ mice have dramatically shortened life span (Fig. 4a). Interestingly, 100% *Brca1*^*−/−*^*;Trp53bp1*^*−/−*^ mice succumbed to thymic lymphoma within 7 months while none of the *Trp53bp1*^*−/−*^ mice in the cohort were dead during this period (Fig. 4a, b), which is a stronger phenotype than that observed in *Brca1*^*ΔC/ΔC*^*;Trp53bp1*^*−/−*^ mice. Most lymphomas were CD4 and CD8 double positive, suggesting that transformation occurred at this stage of T lymphocytes development (Fig. 4c).

In 1-month-old *Brca1*^*−/−*^*;Trp53bp1*^*−/−*^ mice when lymphomas were not developed or 3-month-old *Brca1*^*−/−*^*;Trp53bp1*^*−/−*^ mice when lymphomas were not developed in most mice, T lymphocytes in the thymus and spleen as well as B lymphocytes in the spleen were indistinguishable from those in WT mice (Figs. 4e, S1, and S2). However, in *Brca1*^*−/−*^*;Trp53bp1*^*−/−*^ mice that were viable with no obvious symptoms at 5 months, early stage thymic lymphomas containing CD4 and CD8 double positive lymphocytes were frequently observed (Figs. S1 and S2). Histological examinations revealed that cell organization of the thymus was completely disrupted (Fig. S3). These observations suggested that thymic lymphomas in *Brca1*^*−/−*^*;Trp53bp1*^*−/−*^ mice initiated by expansion of CD4 and CD8 double positive T lymphocytes. In agreement with this observation, the T lymphocytes in the spleen were predominantly double positive for CD4 and CD8 (Fig. S2). In addition, CD19 positive B lymphocytes were dramatically reduced in the spleen (Fig. S2). Cell organization of the spleen was also severely disrupted (Fig. S3). These observations together suggested that the lymphoma cells had massively infiltrated the spleen and replaced the original cells in the spleen. The infiltration of the lymphoma cells was also found in other organs such as kidney, despite that the overall structure of the kidney was not altered (Fig. S3).

The thymic lymphoma phenotype in *Brca1*^*−/−*^*;Trp53bp1*^*−/−*^ mice is strikingly similar to the phenotype of several DNA damage response gene KO mice, such as *Trp53* KO mice and *Atm* KO mice [20–23]. Unlike lymphomas from *Atm* KO mice that have low TCRβ expression, lymphomas from *Brca1*^*−/−*^*;Trp53bp1*^*−/−*^ mice were highly positive for TCRβ (Fig. 4d). Unlike *Atm* KO mice, there was no defect in TCRβ rearrangement in 1-month-old *Brca1*^*−/−*^*;Trp53bp1*^*−/−*^ mice when lymphomas were not developed (Fig. 4f). Therefore, thymic lymphomas in *Brca1*^*−/−*^*;Trp53bp1*^*−/−*^ mice are unlikely caused by ATM-dependent signaling defects.

### BRCA1-53BP1 DKO lymphomas have unique characteristics of genomic instability

**Fig. 5:**
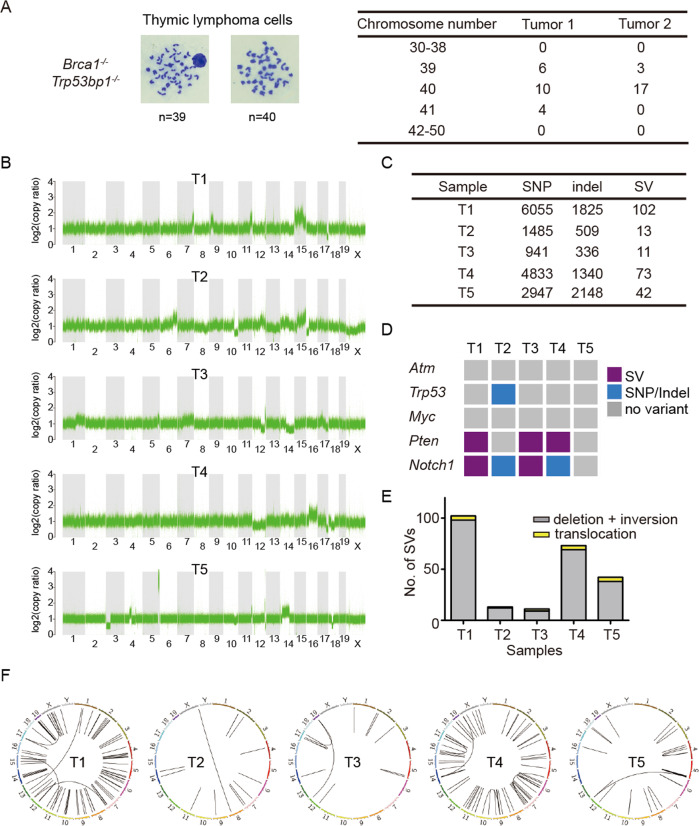
BRCA1-53BP1 DKO lymphomas have unique characteristics of genomic instability. **a** Representative metaphase spreads of lymphoma cells from 2 BRCA1-53BP1 DKO mice. For each lymphoma, chromosome numbers were counted in 20 metaphase spreads and summarized. **b** Copy number profiling in five lymphoma samples by whole-genome sequencing analysis. Log2-transformed copy ratios are plotted to show gain/loss in genomic regions. **c** Summary of genomic instabilities identified in five sequenced lymphoma samples. Genomic instabilities include single nucleotide polymorphisms (SNP), small insertions and deletions (Indel), and large structural variations (SV). **d** Mutational status in genes known to be correlated with thymic lymphoma onset. **e** Distributions of different types of structural variants in each lymphoma sample. Bar graph plotted the number of intra-chromosomal changes (deletion and inversion) and inter-chromosomal changes (translocation). **f** Circos plots of lymphoma samples showing locations of structural variations.

Genomic instability often leads to aneuploidy. In contrast to *Trp53* KO mice [24], no aneuploidy was observed in lymphomas from *Brca1*^*−/−*^*;Trp53bp1*^*−/−*^ mice (Fig. 5a), suggesting that p53 signaling was normal. To provide insights into genomic features of these lymphomas, we performed whole-genome sequencing (WGS) of five individual lymphoma samples and their corresponding liver samples as germ line control. Consistent with cytological studies, no global chromosome number changes were identified in any of the lymphoma samples (Fig. 5b). Each lymphoma harbored a few fragment gain or loss, but no common altered regions across all samples were found (Fig. 5b). Unlike lymphomas from *Atm* KO mice, trisomy chromosome 15 was not observed (Fig. 5b).

Many single nucleotide polymorphisms (SNPs), small insertions or deletions (Indels), and structural variations (SVs) were identified in each lymphoma (Fig. 5c), reflecting genomic instability in these tumors. No genomic alternation was identified at *Atm* or *Trp53* gene loci except one SNP with unknown pathogenic property in *Trp53* gene (Fig. 5d), further suggesting that ATM and p53 signaling is intact. Unlike lymphomas from *Atm* KO mice, there were no alterations of *Myc* oncogene either (Fig. 5d). However, similar to lymphomas from *Atm* KO or *Trp53* KO mice, in which deletions of tumor suppressor gene *Pten* and mutations/amplifications of *Notch1* oncogene are often identified, the majority (80%) of the sequenced lymphomas from *Brca1*^*−/−*^*;Trp53bp1*^*−/−*^ mice contained SVs at *Pten* and/or *Notch1* locus (Fig. 5d). This suggests that although genomic instability can originate from different signaling defects at different developmental stages of T cells, loss of functional PTEN and NOTCH1 is often selected to drive thymic lymphoma development.

SVs, including intra-chromosomal large deletions/inversions and inter-chromosomal translocations, are deleterious genomic alterations that are frequently identified in tumors. Different numbers of sVs were found in five lymphoma samples, but no common altered fragments were identified (Fig. 5e). In lymphomas from *Atm* KO mice, inter-chromosomal translocations between chromosome 12 and 14 are most frequently observed. However, they were not present in lymphomas from *Brca1*^*−/−*^*;Trp53bp1*^*−/−*^ mice (Fig. 5f). The above analyses together reveal that genomic instability in *Brca1*^*−/−*^*;Trp53bp1*^*−/−*^ mice likely leads to a unique type of thymic lymphoma with intact ATM and p53 signaling.

### BRCA1-53BP1 DKO cells have increased microhomology-mediated end joining

**Fig. 6:**
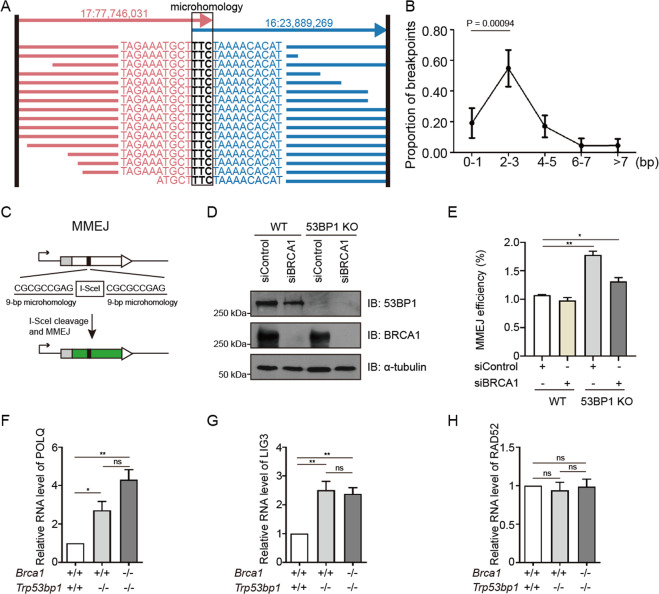
BRCA1-53BP1 DKO cells have increased microhomology-mediated end joining. **a** An example of structural variation with microhomology identified in sequencing analysis. Each row represents one aligned read that supports this translocation contig. Only sequences near the breakpoint joint are shown. The two genomic regions are colored differently in red and blue. The 3-bp microhomology sequence in this breakpoint is marked. **b** Analysis of the microhomology length distribution in structural variation breakpoints. Two-tailed *t*-test was applied to calculate the statistical significance between ratios in different length group. **c** Schematic representation of the MMEJ reporter for analyzing efficiency of DSB repair by MMEJ. **d** Western blotting analyses of BRCA1 and 53BP1 protein in WT and 53BP1 KO U2OS MMEJ-GFP cells after control or BRCA1 knockdown. α-tubulin was used as loading control. **e** Summary of MMEJ efficiency in WT and 53BP1 KO U2OS MMEJ-GFP cells after control or BRCA1 knockdown. Quantification of POLQ (**f**), LIG3 (**g**), and RAD52 (**h**) mRNA levels in MEFs with indicated genotypes. Error bars represent SEM from three independent experiments. *p < 0.05; **p < 0.01; ns not significant.

SVs occur by ligating two broken fragments at distant genomic loci through canonical NHEJ (cNHEJ) or microhomology-mediated end joining (MMEJ) using homologous sequences at breakpoints. Examination of sequence patterns at variation junctions can infer which pathways are utilized for ligation of the two joints. Precise junctions are likely ligated through cNHEJ, while junctions with shared nucleotides between breakpoints (microhomologies) are likely ligated through MMEJ (Fig. 6a) [25, 26]. It has been shown that >80% junctions of SVs in *Brca2* mutant murine brain tumors have no or very short microhomologies (0–1 bp), suggesting that cNHEJ are mainly responsible for the ligation [26]. Interestingly, in lymphomas from *Brca1*^*−/−*^*;Trp53bp1*^*−/−*^ mice, only 20% junctions had no or very short microhomologies (0–1 bp), and most junctions had longer microhomologies (Fig. 6b). In particular, more than 50% junctions had microhomologies of 2–3 bp (Fig. 6b). This observation suggests that SVs in these lymphomas are predominantly ligated through MMEJ.

The above in vivo study prompts that *Brca1*^*−/−*^*;Trp53bp1*^*−/−*^ cells might have increased MMEJ activity. We tested this possibility using U2OS cells stably integrated with an MMEJ reporter. Consistent with role of 53BP1 in suppressing DNA end resection, 53BP1 KO significantly increased the efficiency of MMEJ that requires DNA end resection (Fig. 6c–e). The MMEJ efficiency was also higher in cells deficient for both BRCA1 and 53BP1 than in WT cells (Fig. 6c–e), which is likely the same in *Brca1*^*−/−*^*;Trp53bp1*^*−/−*^ cells. This idea was further supported by the observation that the expression levels of POLQ and LIG3, two key proteins in MMEJ pathway, were significantly increased in *Brca1*^*−/−*^*;Trp53bp1*^*−/−*^ MEFs (Fig. 6f, g).

Besides HR and MMEJ, single-strand annealing (SSA) is another repair pathway that requires DNA end resection. Using U2OS cells stably integrated with an SSA reporter, we have shown before that although 53BP1 KO significantly increases the efficiency of SSA, further depletion of BRCA1 in 53BP1 KO cells reduces the SSA efficiency to a similar level as in WT cells [27]. Therefore, it is likely that *Brca1*^*−/−*^*;Trp53bp1*^*−/−*^ cells do not have altered SSA activity. In agreement with this idea, the expression level of RAD52, the key proteins in SSA pathway, was not changed in *Brca1*^*−/−*^*;Trp53bp1*^*−/−*^ MEFs (Fig. 6h). Therefore, MMEJ, but not SSA, is specifically activated in these cells.

### BRCA1-53BP1 DKO cells have defective G2/M cell cycle checkpoint

**Fig. 7:**
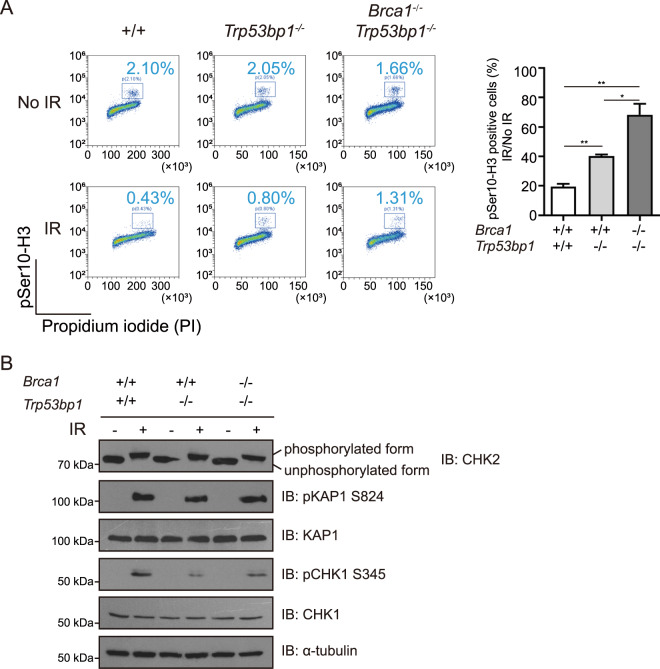
BRCA1-53BP1 DKO cells have defective in G2/M cell cycle checkpoint. **a** Representative flow cytometry analyses of mitotic cells defined as 4 N cells positive for histone H3 phosphorylation (S10) in MEFs with indicated genotypes 1 h after 5 Gy IR exposure. Error bars represent SEM from three independent experiments. *p < 0.05; **p < 0.01. **b** Western blotting analyses of CHK2, KAP1 phosphorylation (S824), KAP1, CHK1 phosphorylation (S345), and CHK1 in MEFs with indicated genotypes 1 h after 10 Gy IR exposure. α-tubulin was used as loading control.

Besides DNA repair, both BRCA1 and 53BP1 participate in DNA damage signaling. Indeed, *Trp53bp1*^*−/−*^ MEFs had defective G2/M cell cycle checkpoint (Fig. 7a). Although *Brca1*^*−/−*^ MEFs could not be obtained for examination, *Brca1*^*−/−*^*;Trp53bp1*^*−/−*^ MEFs displayed more severe defect in G2/M cell cycle checkpoint than *Trp53bp1*^*−/−*^ MEFs (Fig. 7a). It is possible that the loss of BRCA1 and 53BP1 synergistically contributes to the G2/M cell cycle checkpoint defect.

ATM-dependent CHK2 activation is the major signaling pathway to activate G2/M cell cycle checkpoint after DNA damage. However, no defect in IR-induced CHK2 phosphorylation was observed in *Brca1*^*−/−*^*;Trp53bp1*^*−/−*^ MEFs (Fig. 7b). IR-induced phosphorylation of ATM substrate KAP1 was normal as well (Fig. 7b). These data are highly consistent with the above observations that the lymphoma and T cells in *Brca1*^*−/−*^*;Trp53bp1*^*−/−*^ mice had no defects similar to those in *Atm* KO mice (Fig. 4c–f). ATR-dependent CHK1 activation also contributes to DNA damage-induced G2/M cell cycle checkpoints [28]. Interestingly, both *Trp53bp1*^*−/−*^ MEFs and *Brca1*^*−/−*^*;Trp53bp1*^*−/−*^ MEFs had defects in IR-induced CHK1 phosphorylation (Fig. 7b). This observation suggests that defective ATR-CHK1 signaling is likely the major reason behind the G2/M cell cycle checkpoint defect, which might be one of causes for lymphoma development.

### 53BP1 expression is decreased in cancers with silenced BRCA1 expression

BRCA1 is frequently mutated in familial breast and ovarian cancers. Previous studies have shown that 53BP1 loss is associated with triple-negative and BRCA1 mutated breast cancers [29]. Besides mutations, BRCA1 is silenced through promoter hypermethylation in some breast or ovarian cancers, causing diminished BRCA1 expression level similar to the BRCA1 status in *Brca1*^*−/−*^ mice. To test if 53BP1 loss can also promote the growth of tumors with diminished BRCA1 expression, we ranked breast cancer samples in TCGA database based on BRCA1 expression levels and compared 53BP1 expression levels between BRCA1-low (lowest 10%) and BRCA1-high (highest 10%) samples. Interestingly, 53BP1 expression was significantly lower in BRCA1-low than in BRCA1-high samples (Fig. S4A). Similar phenomenon was observed in ovarian cancer samples in TCGA database (Fig. S4B). We went on to test if the positive correlation between BRCA1 and 53BP1 expression levels in breast cancer samples are influenced by prognostic molecular profiling factors. We first separated breast cancer samples into two groups based on estrogen receptor (ER) expression status (positive or negative) and ranked the samples in each group based on BRCA1 expression levels. 53BP1 expression was significantly lower in BRCA1-low than in BRCA1-high samples in both groups (Fig. S4C), suggesting that ER expression did not affect the positive correlation between BRCA1 and 53BP1 expression levels. Similarly, progesterone receptor (PR) or HER2 expression did not have any effect either (Fig. S4D, E). This correlation was also significant for triple-negative breast cancer samples, which were negative for ER, PR, and HER2 (Fig. S4F). Therefore, the positive correlation between BRCA1 and 53BP1 expression levels was independent of the above prognostic molecular profiling factors. These results suggest that 53BP1 loss might promote the development of cancers with silenced BRCA1 expression.

## Discussion

### Domains of BRCA1 determine the ability of 53BP1 KO to rescue HR-related defects

**Fig. 8:**
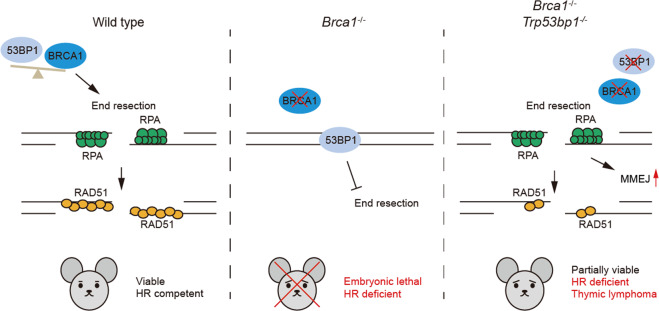
Working model: 53BP1 loss partially rescue embryonic lethality of BRCA1 total knockout mice without restoring HR. BRCA1 is critical for DSB repair by homologous recombination (HR). In WT mice, BRCA1 promotes DNA end resection by removing 53BP1 from DSB ends and facilitates HR repair (left panel). 53BP1 fails to be removed in BRCA1 total KO mice (*Brca1*^*−/−*^) and occupies DSB ends. DNA end resection is inhibited, which results in HR deficiency and embryonic lethality of BRCA1 total KO mice (middle panel). Loss of 53BP1 lifts the barrier for DNA end resection in BRCA1 total KO mice. HR is not restored but microhomology-mediated end joining (MMEJ) is increased. *Brca1*^*−/−*^*;Trp53bp1*^*−/−*^ mice are partially viable, but they have severe genomic instability and develop thymic lymphomas (right panel).

In this study, we have demonstrated that 53BP1 KO can partially rescue embryonic lethality of BRCA1 total KO mice that are homozygous for a *bona fide Brca1* null allele. Unlike *Brca1*^*Δ11/Δ11*^*;Trp53bp1*^*−/−*^ and *Brca1*^*Δ2/Δ2*^*;Trp53bp1*^*−/−*^ mice but similar to *Brca1*^*ΔC/ΔC*^*;Trp53bp1*^*−/−*^ mice, our study in *Brca1*^*−/−*^*;Trp53bp1*^*−/−*^ mice suggests that 53BP1 KO can partially rescue embryonic lethality but not HR deficiency, genomic instability, or PARPi sensitivity caused by complete BRCA1 loss. As a result of severe genomic instability, all *Brca1*^*−/−*^*;Trp53bp1*^*−/−*^ mice die of thymic lymphoma within 7 months (Fig. 8). Both BRCA1Δ11 and BRCA1Δ2 proteins have BRCT domains that facilitate their localization to DNA damage sites and coiled coil domains that interact with PALB2, but these domains are absent in BRCA1ΔC proteins or the remaining peptides in *Brca1*^*−/−*^ cells, suggesting that these domains are important for 53BP1 KO to rescue HR-related deficiency.

BRCA1 forms a heterodimer with BARD1 through its N-terminal RING domain, which is required for BARD1’s protein stability [18, 19]. In both *Brca1*^*Δ2/Δ2*^*;Trp53bp1*^*−/−*^ and *Brca1*^*−/−*^*;Trp53bp1*^*−/−*^ cells, the absence of RING domain leads to dramatic reduction of BARD1 levels. Although not examined, it is likely that BARD1 levels are also diminished in *Brca1*^*ΔC/ΔC*^*;Trp53bp1*^*−/−*^ cells, given the dramatic reduction of BRCA1ΔC protein levels [16]. Since 53BP1 KO rescues HR-related deficiency in *Brca1*^*Δ2/Δ2*^*;Trp53bp1*^*−/−*^ cells, but fails to do so in *Brca1*^*ΔC/ΔC*^*;Trp53bp1*^*−/−*^ or *Brca1*^*−/−*^*;Trp53bp1*^*−/−*^ cells, it is likely that the BRCT and/or coiled coil domains of BRCA1 are required for 53BP1 KO to rescue HR-related deficiency in the absence of BARD1. BARD1 can also localize to early DNA damage sites independent of BRCA1’s BRCT domains [30]. Recent studies have shown that BARD1 can directly interact with RAD51 [31]. It will be interesting to investigate if restoration of BARD1 protein level can alleviate the requirement of BRCA1’s BRCT and/or coiled coil domain for 53BP1 KO to rescue HR-related deficiency.

### HR deficiency can be partially uncoupled from embryonic lethality

Since BRCA1 is critical for HR, it is generally believed that HR deficiency is the major cause of embryonic lethality of *Brca1*^*−/−*^ embryos. Although unable to restore HR deficiency, 53BP1 KO partially rescued embryonic lethality of *Brca1*^*−/−*^ embryos and no abnormality was observed in the viable *Brca1*^*−/−*^*;Trp53bp1*^*−/−*^ mice until they die of thymic lymphoma. Together with the previous study using *Brca1*^*ΔC/ΔC*^*;Trp53bp1*^*−/−*^ mice [16], our study has suggested that HR deficiency can be partly compatible with embryonic development in some BRCA1 mutant mice. In agreement with this idea, BRCA1 BRCT mutant mice (*Brca1*^*S1598F/S1598F*^) can survive, have HR deficiency, but appear normal before tumor onset [32]. Similar to BRCA1 mutants, KO mice of several Fanconi anemia complementation group proteins are viable but have HR defects [33]. Therefore, HR deficiency can be partially uncoupled with embryonic lethality in mice.

Compared with *Brca1*^*Δ11/Δ11*^ cells, *Brca1*^*−/−*^*;Trp53bp1*^*−/−*^ cells are slightly less sensitive to PARPi (Fig. 3b). Since PARPi sensitivity strongly correlates with HR efficiency, this result suggests that HR efficiency is slightly higher in *Brca1*^*−/−*^*;Trp53bp1*^*−/−*^ cells than in *Brca1*^*Δ11/Δ11*^ cells. Although *Brca1*^*−/−*^ cells cannot be obtained for analysis due to cell lethality, *Brca1*^*−/−*^ cells should theoretically have similar or even lower HR efficiency than *Brca1*^*Δ11/Δ11*^ cells. Therefore, we speculate that HR efficiency is slightly higher in *Brca1*^*−/−*^*;Trp53bp1*^*−/−*^ cells than in *Brca1*^*−/−*^ cells, which suggests that *53bp1* KO can mildly rescue the HR defects in *Brca1*^*−/−*^ cells.

A previous study has shown that RNF168 can also load PALB2 to DNA damage sites independent of BRCA1 [34]. A recent report has also suggested that RNF168-dependent PALB2 loading is required for the viability of BRCA1 haploinsufficient mice [35]. Since 53BP1 KO has lifted the barrier for DNA end resection in *Brca1*^*−/−*^ cells, it is possible that RNF168 can load PALB2 to DNA damage sites in *Brca1*^*−/−*^*;Trp53bp1*^*−/−*^ MEFs and mildly rescue the HR defects in *Brca1*^*−/−*^ cells. Although we did not directly test this idea, we speculate that even if RNF168 can load PALB2 to damaged sites in these cells, this pathway has limited contribution to HR in these cells, given the severe HR deficiency in *Brca1*^*−/−*^*;Trp53bp1*^*−/−*^ MEFs.

### MMEJ potentially contribute to the survival of BRCA1-53BP1 DKO embryos

Besides HR, MMEJ and SSA are two repair pathways that also require DNA end resection. Reporter assays suggest that the repair efficiency of MMEJ, but not SSA, is activated in *Brca1*^*−/−*^*;Trp53bp1*^*−/−*^ cells. These observations are consistent with previous reports that HR-deficient tumor cells have increased POLQ expression and rely on MMEJ for survival [36–38]. Previous studies have revealed that although either FANCD2 KO or POLQ KO mice are viable, FANCD2 and POLQ DKO mice are largely embryonic lethal, suggesting that the loss of both HR and MMEJ leads to embryonic lethality. Since *Brca1*^*−/−*^*;Trp53bp1*^*−/−*^ cells have severe HR deficiency, elevated MMEJ activity in these cells should reflect the significant contribution of this pathway to the survival of these embryos. As MMEJ is highly mutagenic, it is possible that *Brca1*^*−/−*^*;Trp53bp1*^*−/−*^ embryos survive at the cost of having increased genomic instability and eventually develop thymic lymphoma.

BRCA1 is also required for protecting replication forks after replication stress [39]. However, 53BP1 KO rescued embryonic lethality but not replication fork protection defects caused by different BRCA1 mutants in previous studies [15, 16, 39]. Therefore, it is unlikely that 53BP1 KO rescued replication fork protection defects in *Brca1*^*−/−*^*;Trp53bp1*^*−/−*^ cells.

Although *Brca1*^*−/−*^*;Trp53bp1*^*−/−*^ mice are born at reduced Mendelian ratio, *Brca1*^*−/−*^*;Trp53bp1*^*−/−*^ embryos at 13.5 dpc can be obtained with normal Mendelian ratio. This suggests that some mice die during late embryonic development, when hematopoietic organs are developed and hematopoietic stem cells transit from fetal liver to fetal bone marrow [40]. Recent studies have shown that BRCA1 is important for hematopoiesis [41, 42]. Therefore, hematopoietic defects might account for the death of some *Brca1*^*−/−*^*;Trp53bp1*^*−/−*^ embryos during late embryonic development. The detailed mechanism requires further investigation.

### BRCA1-53BP1 DKO mice develop a unique type of thymic lymphoma

Although *Brca1*^*−/−*^*;Trp53bp1*^*−/−*^ mice are viable, they have severe genomic instability and eventually develop thymic lymphoma within 7 months. These lymphomas are distinct from those from *Atm* or *Trp53* KO mice because BRCA1, ATM, and p53 have different roles in DNA damage repair and signaling. ATM has a minor role in HR that is distinct from BRCA1. Unlike BRCA1, ATM also functions in NHEJ that is required for V(D)J development in lymphocytes. This explains why *Atm* KO mice, but not in *Brca1*^*−/−*^*;Trp53bp1*^*−/−*^ mice, have defective TCRβ rearrangement.

*TP53* mutations are frequently found in BRCA1-associated human breast cancers [43, 44]. Therefore, the absence of *Trp53* mutations or SVs in lymphomas from *Brca1*^*−/−*^*;Trp53bp1*^*−/−*^ mice is surprising. *TP53* mutations often occur late in development to offer growth advantage of tumors that originate from other genetic alterations [45]. It is possible that the intrinsic G2/M cell-cycle checkpoint defects in *Brca1*^*−/−*^*;Trp53bp1*^*−/−*^ cells alleviate the requirement for *Trp53* mutations and offer growth advantage to allow rapid development of thymic lymphomas. Therefore, the combination of defective HR, elevated MMEJ, and compromised cell cycle checkpoint leads to the development of a unique type of thymic lymphoma in *Brca1*^*−/−*^*;Trp53bp1*^*−/−*^ mice.

## Materials and methods

### Mice

*Brca1*^*flox/flox*^ mice (NCI Mouse Repository, 01XB8, mixed background of FVB and 129) were mated with *Ddx4-cre* mice (The Jackson Laboratory, 006954, obtained in FVB background and backcrossed to C57 background) to obtain *Brca1*^*Δ5−*^^*13/+*^ mice. They were mated with *Trp53bp1*^*−/−*^ mice (The Jackson Laboratory, 006495, mixed background of C57 and 129) for two generations to obtain *Brca1*^*Δ5−*^^*13/+*^*;Trp53bp1*^*−/−*^ mice. These mice were subsequently intercrossed to generate mice used in this study. All mice studies were approved by the Zhejiang University Animal Care and Use Committee.

### Cell culture

MEF and U2OS cells were maintained in DMEM supplemented with 10% fetal bovine serum and 1% penicillin and streptomycin. Mouse embryonic stem cells (ES cells) were cultured in gelatin-coated plates in DMEM medium with 15% FBS, 1% penicillin and streptomycin, 1× nonessential amino acids, 1× l-glutamine, 10 ng/ml LIF (Santa Cruz), and 0.1 mM β-mercaptoethanol.

To isolate MEFs, 13.5 dpc mouse embryos were minced with surgical blades and then dissociated in 1× TrpLE (GIBCO) at 37 °C for 30 min in a 15 ml pipette with occasional shaking. Liberated cells were plated and cultured. MEFs at passage 2 were transfected with SV40 T-antigen plasmids for immortalization.

To generate ES cells, 3.5 dpc blastocysts were isolated from naturally mated females by flushing the uterus with M2 medium (Sigma) and were cultured on irradiated MEF feeders in ES cell medium. Seven days later, blastocyst outgrowth was trypsinized and cells were plated on irradiated MEF feeders for expansion. Feeders were gradually removed and ES cells were cultured in gelatin-coated plates for experiments.

WT MEFs and ES cells were generated from embryos obtained by intercross of *Brca1*^*+/−*^ mice. *Trp53bp1*^*−/−*^ and *Brca1*^*−/−*^*;Trp53bp1*^*−/−*^ MEFs and ES cells were generated from embryos obtained by intercross of *Brca1*^*+/−*^*;Trp53bp1*^*−/−*^ mice.

### RNA interference

The siRNA target sequences were as follows: Control, UUCUCCGAACGUGUCACGUdTdT; BRCA1 #1, CAGCUACCCUUCCAUCAUAdTdT. siRNA transfection was performed using Lipofectamine 3000 transfection reagent (Invitrogen) according to the manufacturer’s instructions.

### Generation of 53BP1 KO cells

DR-GFP U2OS and MMEJ-EGFP U2OS cell lines were gifts from Jeremy Stark (City of Hope) and Xiaohua Wu (Scripps Research Institute), respectively. 53BP1 KO was generated in these cell lines by CRISPR/Cas9 technology. 53BP1 guide RNA (5′-CATAGCAGAACAGTCCAGCA-3′) was cloned into the PX459 V2.0 (gifts from Feng Zhang, Addgene 62988) plasmids. Twenty-four hours after transfection of the PX459 V2.0-53BP1 guide RNA, cells were cultured in the presence of puromycin (2 μg/ml) for 48 h and plated to isolate individual clones. KO cells were validated by western blotting.

### Antibodies

Anti-BRCA1 [46] and anti-53BP1 [47] were gifts from Xiaochun Yu (City of Hope), anti-BARD1 [48] was a gift from Richard Baer (Columbia University). The follow antibodies were purchased: anti-γH2AX (Abcam, ab81299), anti-phospho-KAP1 S824 (Abcam, ab70369), anti-phospho-RPA2 (S4/S8) (Bethyl, A300-254A), anti-RAD51 (Santa Cruz, sc-8349), anti-CHK1 (Santa Cruz, sc-8408), anti-phospho-CHK1 S345 (CST, #2348S), anti-phospho-Histone H3 S10 (CST, #9701S), anti-RPA2 (CST, #2208S), anti-KAP1 (Sangon Biotech, D155285), anti-BrdU (BD biosciences, 347580), anti-CHK2 (BD biosciences, 611571), FITC-anti-mouse CD4 (BD biosciences, 557307), PE-anti-mouse CD8a (BD biosciences, 553032), and PE-anti-mouse TCRβ chain (BD biosciences, 561081).

### Western blotting

Cells were lysed with RIPA buffer (50 mM Tris-HCl, 150 mM NaCl, 5 mM EDTA, 1% Nonidet P-40, 0.1% SDS, 0.5% sodium deoxycholate) containing 1 μg/ml of phosphatase inhibitor on ice for 15 min. Lysates were centrifuged at 16900 × *g* for 5 min to remove cellular debris. Samples were boiled after the addition of loading buffer. Proteins were separated by SDS–PAGE, transferred to PVDF membranes. The membrane was blocked with 5% milk for 10 min and blotted with indicated antibodies overnight, washed three times with TBST buffer, and blotted with HRP-linked goat anti-rabbit or goat anti-mouse secondary antibodies (Jackson ImmunoResearch) for 1 h. After three washes with TBST buffer, blots were analyzed by enhanced chemiluminescence system.

### Immunofluorescent staining

MEFs cultured on coverslips were treated with 10 Gy IR and recovered for 6 h under the same culture condition. Cells were then washed with PBS once, pre-extracted with 0.5% Triton X-100 for 1 min, and fixed with 4% paraformaldehyde for 10 min at room temperature. Cells were then incubated with primary antibody for 1 h at room temperature. After washing with PBS for twice, cells were incubated with Alexa Fluor 488/594 labeled secondary antibody (Jackson ImmunoResearch) for 30 min at room temperature. Cells were subsequently stained with Hoechst 33342 and visualized using a fluorescence microscope (Eclipse Ti2; Nikon).

### Single-stranded DNA detection at DNA damage sites

MEFs were grown in culture medium containing BrdU (10 μM) for 24 h. Cells were then treated with 10 Gy IR and recovered for 1 h under the same culture condition. Cells were subjected to immunofluorescent staining using an anti-BrdU antibody.

### Metaphase spreads analysis

Cells were treated with or without PARPi olaparib (1 μM) overnight and arrested with colcemid (0.2 μg/ml) for 2 h. Cells were collected, resuspended in 75 mM KCl and incubated at room temperature for 8 min. Cells were then fixed with methanol-acetic acid (3:1 (v/v)) and dropped onto glass slides. DNA were stained with 5% Giemsa for 5 min. Chromosome numbers and chromosomal aberrations were counted. Twenty metaphases were analyzed for cells of each genotype.

### Plasmid HR reporter assay

DR-GFP plasmids were gifts from Maria Jasin (Memorial Sloan Kettering Cancer Center). MEFs were transfected with DR-GFP plasmids or plasmids expressing an intact GFP protein. Forty-eight hours after transfection, cells were infected with adenovirus expressing I-SceI. After 24 h, GFP positive cells were analyzed by flow cytometry using a Beckman CytoFLEX LX Analyzer. HR efficiency were normalized by transfection efficiency marked by GFP only cells.

### Integrated HR and MMEJ reporter assay

DR-GFP U2OS and MMEJ-EGFP U2OS cell lines were transfected with indicated siRNAs and infected with adenovirus expressing I-SceI. Cells were recovered for 48 h after infection and were analyzed by flow cytometry.

### Cell viability assay

A total of 5 × 10^4^ ES cells or 3 × 10^3^ U2OS cells were plated in each well of 6-well plates. After 24 h, cells were treated with the indicated concentration of PARPi olaparib and medium was changed every day. After incubation for 7 days, the surviving cells were assessed with CCK8 reagent (DOJINDO) following the manufacturer’s instructions.

### Lymphocyte analysis

Single-cell suspensions were prepared from thymus, lymphoma or spleen from mice of the indicated genotypes. A total of 1 × 10^5^ cells were stained using fluorescence-conjugated antibodies as indicated. Lymphocyte populations were analyzed by flow cytometry.

### G2/M cell-cycle checkpoint analysis

MEF cells were exposed to 5 Gy ionizing radiation or without treatment. After 1 h of recovery, cells were fixed with cold 70% ethanol and stained with anti-phospho-Histone H3 S10 antibody. Cells were incubated with FITC-conjugated goat–anti-rabbit secondary antibody. Cells were then treated with RNase A and then incubated with propidium iodide. Samples were analyzed by flow cytometry.

### RNA extraction, reverse transcription, and quantitative RT-PCR

RNA extraction was performed using Trizol (Beyotime Biotechnology) according to the manufacturer’s instructions. Reverse transcription was performed with PrimeScript RT Reagent Kit (Takara). Quantitative RT-PCR was performed using TB Green Premix Ex Taq (Takara). The oligonucleotides were as follows: LIG3 (5′-TATGGGCAAGGGAGCAAAGG-3′ and 5′-GATCTCCCACACAGCAGCTT-3′), RAD52 (5′-CCTATCATGAGGACGTGGGC-3′ and 5′-TGATGTCCTTAGCCCCCTGA-3′), and POLQ (5′-CGTTCTCGGGAGATGGTGAT-3′ and 5′- GAGGAGAACTGTCCCGTTGG-3′).

### Hematoxylin and eosin (H&E) staining

Tissues were fixed in 4% paraformaldehyde for 24 h, dehydrated, and embedded in paraffin. Paraffin sections of 5 μm were stained with H&E.

### WGS and bioinformatics analysis

40× paired-end 150 bp WGS was performed for lymphoma and corresponding liver samples using Illumina NovaSeq. Raw sequencing data were aligned to GRCm38 reference genome by Burrows–Wheeler Aligner mem algorithm (BWA-mem v 0.7.12-r1039) with default parameters [49]. After alignment, reads were sorted by samtools (version 1.1) and duplicated reads were removed by Picard (v 2.0.1) [50] (“Picard Toolkit.” 2019. Broad Institute, GitHub Repository.). To identify SVs with breakpoint sequence pattern, a local-assembly tool SvABA (v 1.1.0) was applied with default parameters [51]. Somatic SVs identified were then filtered using SvABA default filters with an addition condition: no secondary alignments are associated with this contig fragment (SUBN = 0). SV joint sequences were obtained from the filtered SvAVA output. To analyze the microhomology status near SV breakpoints, the length of each microhomology was extracted and the distribution of different length groups (0–1, 2–3, 4–5, 6–7 nt, and more than 7 nt) was calculated. SNPs and small indels were called independently by GATK toolkit (v 4.0.11.0) and SvABA [52]. Variants that were identified in both GATK and SvABA, as well as with more than five supporting reads, were kept as true SNPs/indels. Circos (v 0.69.6) was applied to generate the Circos plots [53]. GATK toolkit was also utilized to calculate copy ratios in tumor samples with their germ line controls. To plot the genome wide copy ratio coverage, bin size was set at 1000 bp. To examine the relationship between BRCA1 and 53BP1 expression, gene expression of breast cancer and ovarian cancer tumors were downloaded from TCGA Data Portal and then log2-transformed. Their corresponding prognostic molecular profiling factors were also obtained from TCGA Data Portal. Unless specified, two-tailed unpaired Student’s *t* test was used to evaluate statistical significance for all experiments. The WGS data has been deposited in NCBI SRA database (PRJNA587820).

## Supplementary information

Supplementary figure legends

Figure S1

Figure S2

Figure S3

Figure S4
