# Supplementary material for: 53BP1 loss rescues embryonic lethality but not genomic instability of BRCA1 total knockout mice
Source: Cell Death Differ. 2020 Mar 5;27(9):2552–67. doi: 10.1038/s41418-020-0521-4 (PMC7429965; doi:10.1038/s41418-020-0521-4)
Supplement: Supplementary file 1 — Supplementary figure legends [file 41418_2020_521_MOESM1_ESM.docx]

**Supplementary Figure legends**

Figure S1. Analysis of thymic T lymphocytes from mice of different ages.

Representative flow cytometry analyses of CD4/CD8 surface expression in T lymphocytes in thymi of 1, 3, and 5-month-old WT and BRCA1-53BP1 DKO mice.

Figure S2. Analysis of splenic T and B lymphocytes from mice of different ages.

Representative flow cytometry analyses of CD4/CD8 surface expression in T lymphocytes (left panel) and CD19 surface expression in B lymphocytes (right panel) in spleens of 1, 3, and 5-month-old WT and BRCA1-53BP1 DKO mice.

Figure S3. Histological examinations of tissues from mice of different ages.

Hematoxylin-and-eosin (H&E) staining of thymus, spleen, and kidney from 1, 3, and 5-month-old WT and BRCA1-53BP1 DKO mice. Asterisks indicate the tumor infiltration. Scale bar, 100 μm.

Figure S4. 53BP1 expression is decreased in cancers with silenced BRCA1 expression.

**A-B.** Boxplots of log2-transformed 53BP1 expression in BRCA1 expression high or low breast cancer (**A,** n = 1104) and ovarian cancer (**B,** n = 309) samples. **C-E.** Boxplots of log2-transformed 53BP1 expression in BRCA1 expression high or low breast cancer samples, with different prognostic molecular profiling factors including ER status (**C**), PR status (**D**) and HER2 status (**E**). **F.** Boxplots of log2-transformed 53BP1 expression in BRCA1 expression high or low triple-negative breast cancers (n = 124) samples. Two-tailed t-test was applied to calculate the significance between groups. To define BRCA1 expression levels, the lowest 10% (BRCA1-low) and the highest 10% (BRCA1-high) samples are used for each type.
