# Supplementary figures and images for: 53BP1 loss rescues embryonic lethality but not genomic instability of BRCA1 total knockout mice
Source: Cell Death Differ. 2020 Mar 5;27(9):2552–67. doi: 10.1038/s41418-020-0521-4 (PMC7429965; doi:10.1038/s41418-020-0521-4)

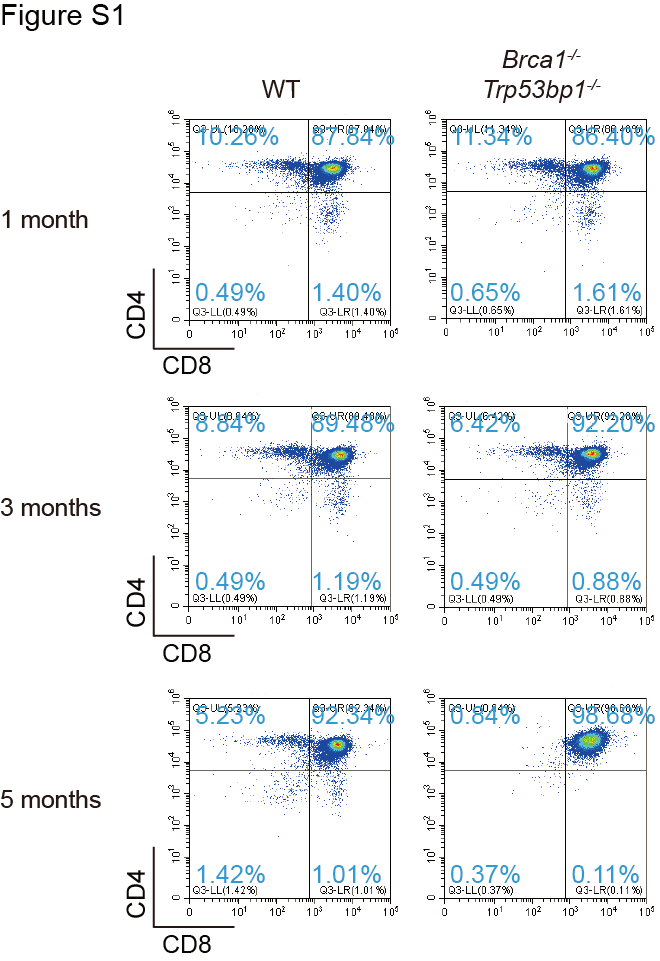

Supplement: Supplementary file 2 — Figure S1 [file 41418_2020_521_MOESM2_ESM.png]

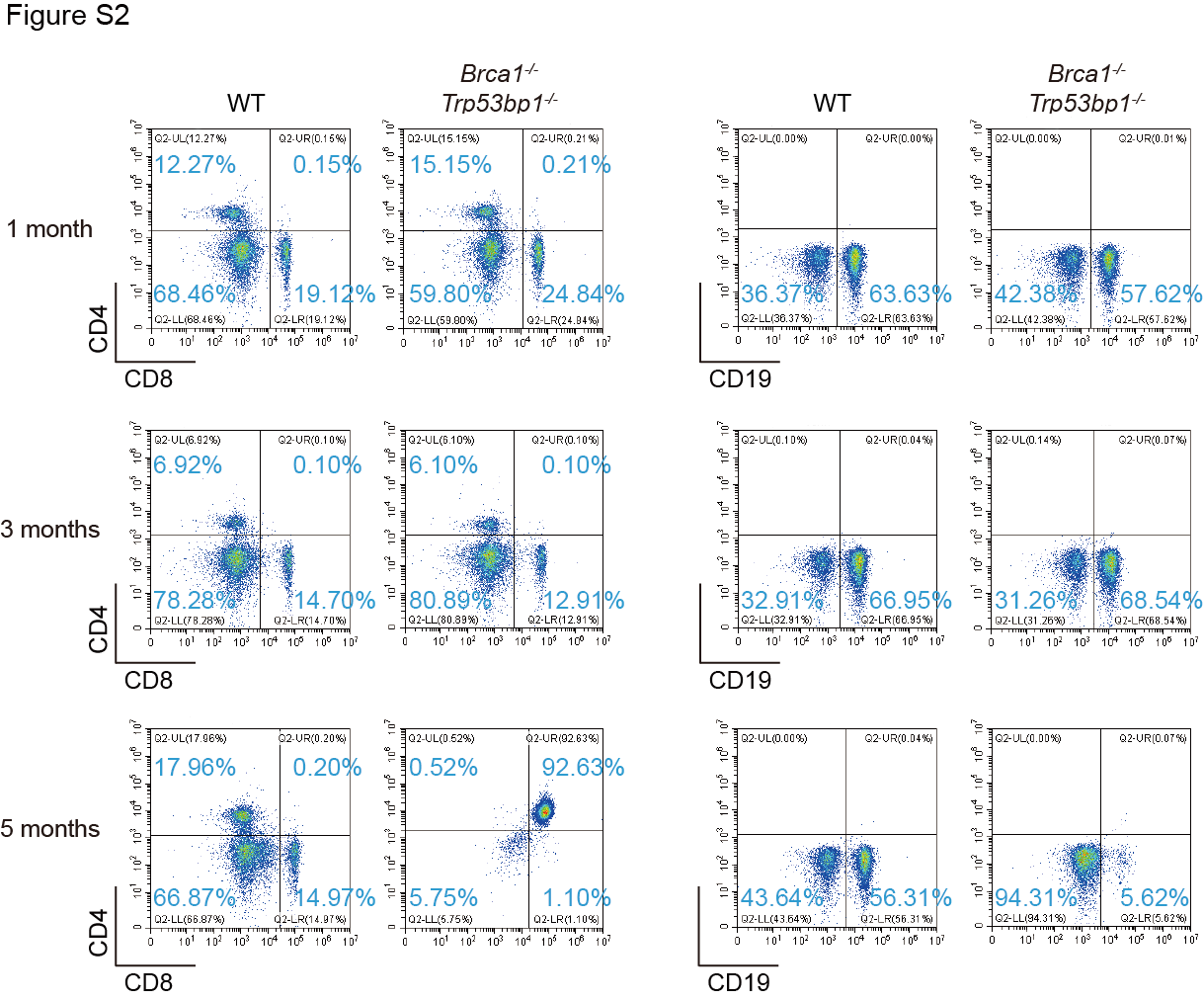

Supplement: Supplementary file 3 — Figure S2 [file 41418_2020_521_MOESM3_ESM.png]

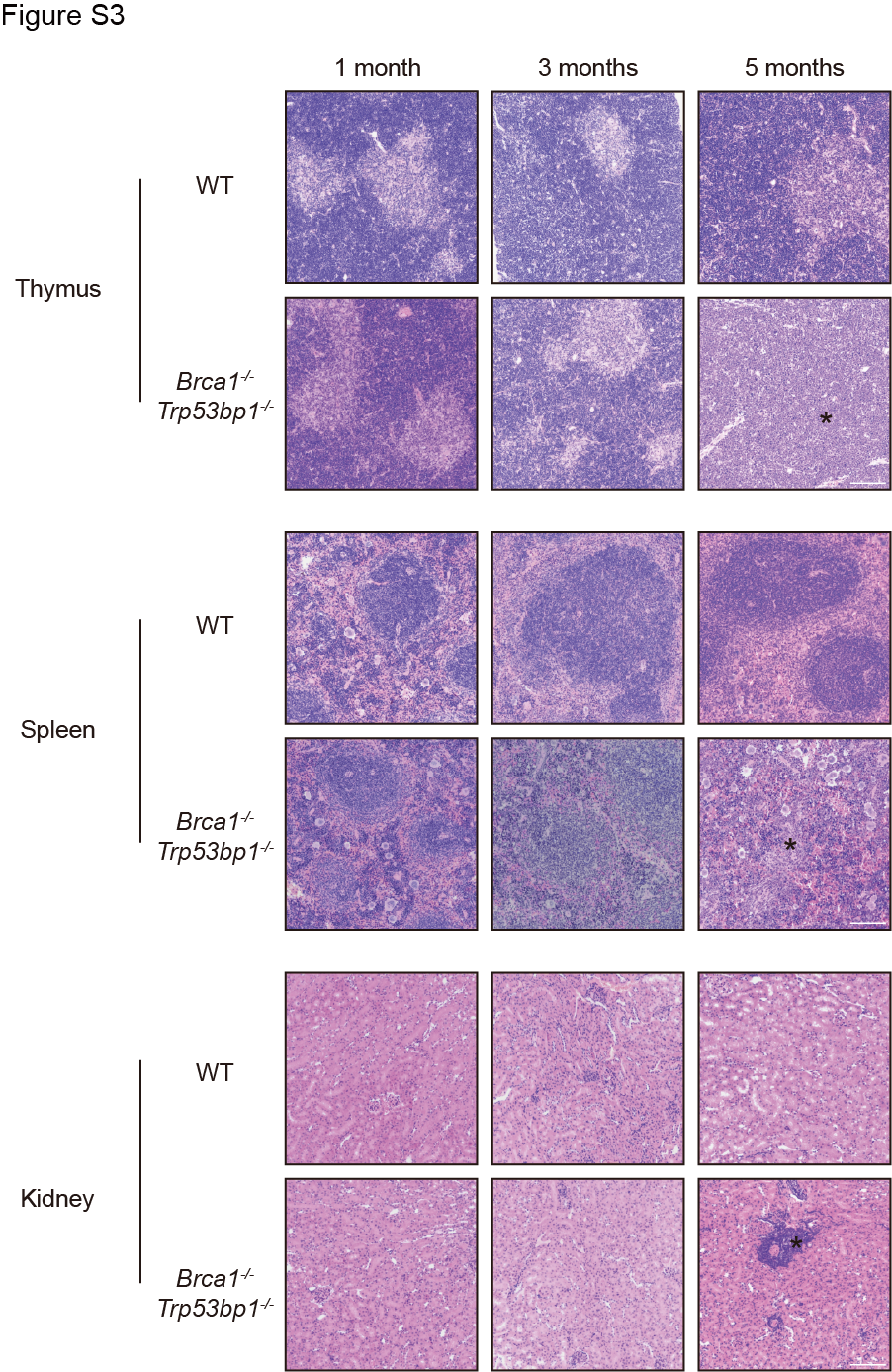

Supplement: Supplementary file 4 — Figure S3 [file 41418_2020_521_MOESM4_ESM.png]

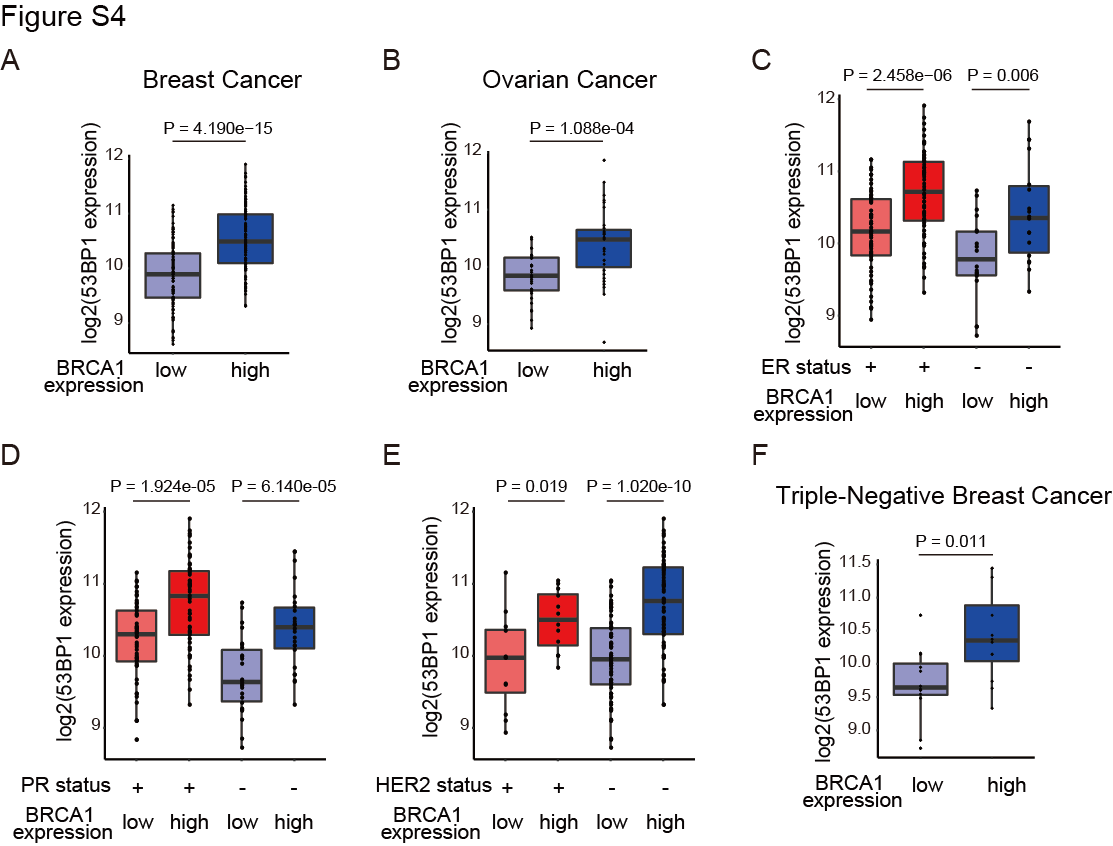

Supplement: Supplementary file 5 — Figure S4 [file 41418_2020_521_MOESM5_ESM.png]
